# The Effect of Cerium Oxide Addition on the Properties and Behavior of Y-TZP

**DOI:** 10.1155/2014/828197

**Published:** 2014-10-29

**Authors:** D. Ragurajan, M. Satgunam, M. Golieskardi

**Affiliations:** Department of Mechanical Engineering, Centre of Ceramic Technology, Universiti Tenaga Nasional, 43000 Kajang, Malaysia

## Abstract

The effects of CeO_2_ addition on the sintering behavior and mechanical properties of Y-TZP have been investigated over a wide sintering regime by pressureless sintering. It has been revealed that small additions of CeO_2_ (0.3–1.0 wt%) to Y-TZP were beneficial in enhancing the mechanical properties and hydrothermal ageing resistance of Y-TZP. Sintered samples were used to evaluate the bulk density, Vickers's hardness, Young's modulus, and fracture toughness of the material. CeO_2_ doped Y-TZPs were sintered at relatively low temperatures (1250°C and 1350°C) retaining high bulk density (>97% of theoretical density) and high Young's modulus (>200 GPa) without sacrificing tetragonal phase stability. The optimum level of dopant was found to be at 0.5 wt% for sintering between 1250°C and 1450°C using the standard 2 h holding time cycle, with sintered body exhibiting excellent combination of properties when compared to the undoped ceramics. In this experiment, the addition of 0.5 wt% recorded a bulk density reading of 5.9 g/cm^3^, Vickers hardness value of 13.2 GPa, Young's modulus value of 211 GPa, and fracture toughness of 6.4 MPam^1/2^, respectively, in a temperature range of 1400–1450°C.

## 1. Introduction

Yttria tetragonal zirconia polycrystal (Y-TZP) ceramics serve as an upcoming material for engineering applications and are also considered important for restoration medicine. The excellent mechanical characteristics such as high strength and fracture resistance are attributed to the ceramic's unique ability known as transformation toughening, a stress-induced phase transformation from tetragonal (t) to monoclinic (m) [1, 2]. The stabilizing effect of yttria (Y_2_O_3_) makes it possible for Y-TZP ceramic to be processed in the metastable tetragonal (t) structure. This is essential since the retention of the (t) phase at ambient temperature allows it to transform to the monoclinic (m) structure under external applied stress [3, 4].

Despite its outstanding properties, Kobayashi et al. [5] discovered that the Y-TZP ceramics suffer a serious limitation for applications near 250°C in moist environment. The findings revealed that the ceramic can suffer a slow, tetragonal to monoclinic phase transformation at the samples surface in a humid atmosphere, followed by microcracking and a major loss in strength known as low temperature degradation (LTD) [6–10]. Since then, experiments have been conducted with Y-TZP in an attempt to understand the basic micromechanisms of the ageing-induced (t)-(m) phase transformation and to suppress the LTD phenomenon [11–19]. Based on research findings, the addition of ceramic oxides (MgO, Al_2_O_3_, ZnO, CaO, and CeO_2_) helps overcome if not prevent the low temperature degradation occurrence in Y-TZP ceramics [9, 20–24]. CeO_2_ is generally used to stabilize the tetragonal phase of zirconia and is also known to increase the sintering of glass ceramics and strength and thermal stability [25]. Ce-TZP in contrast to Y-TZP has complete resistance to LTD and higher toughness. Enhancing the moderate Ce-TZP properties would result in the material becoming a primary candidate for biomedical applications which has the requirements in terms of phase stability and long-lasting characteristics in the human body [26].

Fischer and Stawarczyk found that the Ce-TZP/Al_2_O_3_ nanocomposite offers superior mechanical properties compared to conventional Y-TZP. Also, when compared to conventional Y-TZP the flexural strength is in the same range whereas the fracture toughness increases with the addition of ceria [27]. Results from Mangalaraja et al. and Takano et al. investigating the effect of ceria addition both supported the statement that ceria increases the fracture toughness of Y-TZP [28, 29].

Kuroda et al. [30] have shown that several reactions occur during sintering with the addition of CuO which can be beneficial or detrimental for densification of Y-TZP. More recently, tribological studies on 3 mol% Y-TZP ceramics showed that the addition of 1.8 mol% of CuO led to a reduction of friction coefficient from 0.2 to 0.6 under dry sliding conditions, while another study revealed that the ferromagnetic behaviour of Y-TZP with enhanced coercivity could be achieved by doping 0.3 mol% NiO in the zirconia matrix [31].

The susceptibility of Y-TZP to low temperature degradation (LTD) is a shortcoming for a material that has excellent mechanical properties and biocompatibility. The addition of cerium oxide, CeO_2_, makes the material inert to LTD but is counterbalanced by modest strength and hardness, making its usage in biomedical applications limited and difficult. In order to overcome this limitation, the amount of CeO_2_ addition to Y-TZP is altered along with the sample preparing factors such as sintering methods and temperatures, mixing methods, and the purity of powders used.

## 2. Experimental Techniques

Two commercial type powders were prepared for this experiment: the 3 mol% of yttria-stabilized zirconia powder manufactured by Kyoritsu Ltd., Japan, under the code name of KZ-3YF as the base powder and cerium oxide, CeO_2_, manufactured by Sigma Aldrich, as the dopant. Three different compositions of cerium oxide were mixed with Y-TZP, that is, undoped 0.3 wt%, 0.5 wt%, and 1.0 wt%. The powders were mixed using the wet milling method, using zirconia balls as mixing media and ethanol as the mixing medium. The slurry was oven dried at 60°C sieved, and the powder was uniaxially pressed at 0.3 MPa into discs and rectangular bars. These samples were then sintered under atmospheric condition at a rate of 10°C/min, with temperatures ranging from 1250°C to 1450°C, and a holding time of 2 hours before cooling down to room temperature. The sintering temperatures 1250°C to 1450°C are used for this research as it is sufficient to retain the tetragonal phase of Y-TZP. Besides that, this temperature range is the optimum temperature range to obtain the high mechanical properties of Y-TZP. Sintered disc samples are then used to determine bulk density and Vickers hardness. Disc samples are polished on a polishing machine with grinding papers of different roughness (120, 240, 600, and 800 CC-Cw) and finally are polished using diamond paste of 6 microns and a polishing cloth.

## 3. Characterization

The bulk density of the sintered samples was measured based on Archimedes' principle, using the water immersion method with a standard Mettler Toledo Balance AG204 densimeter. Polished samples were used to determine Vickers's hardness and fracture toughness (*K*_IC_) using Vickers's indentation method. An indentation with a force of 9800 N was applied on the samples and held for 10 seconds. At least three indentations were made and an average value was taken. The fracture toughness was calculated for the measured value obtained using Niihara's [32] formula as stated below:

(1) $$\begin{array}{c} K_{\text{IC}}=\frac{0.016{\left(E/H\right)}^{1/2}}{C^{3/2}}, \end{array}$$

*K*_IC_ being the fracture toughness (MPam^1/2^); *E* is Young's modulus (GPa), *H* the Vickers hardness (GPa), and *c* the crack length (m) measured from the center of indentation. Three measurements were made and the average value was obtained. Young's modulus test was conducted on the rectangular bars by using indentation. The microstructure of the material was evaluated using SEM (scanning electron microscope) whereby the grain structures and grain sizes were determined using the average grain intercept method. The average grain intercept method is used to measure the grain size of a material by drawing randomly positioned line segments on the micrograph, counting the number of times each line segment intersects a grain boundary, and finding the ratio of intercepts to line length.

## 4. Results and Discussion

**Figure 1:**
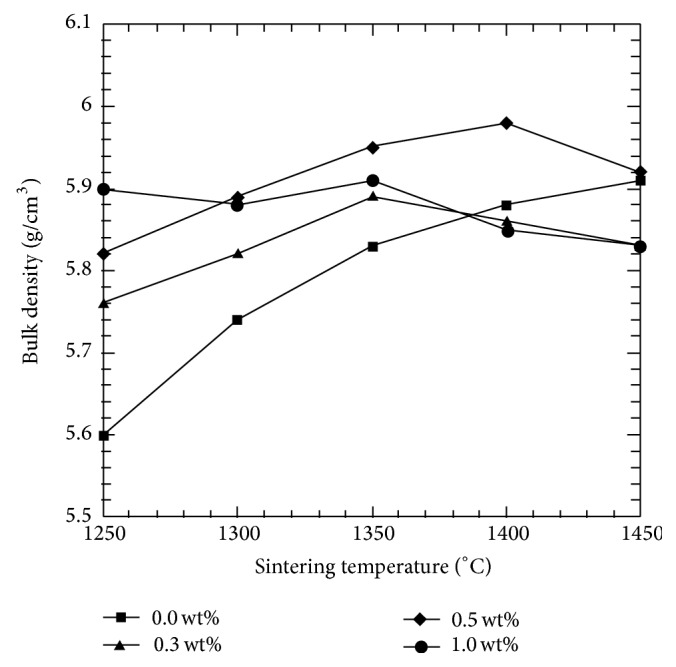
Effect of sintering temperature and CeO_2_ addition to the bulk density of Y-TZP.

Figure 1 shows the influence of various CeO_2_ additions to the bulk density of Y-TZP. The sintering temperature acts as a controlled variable; CeO_2_-Y-TZP samples are sintered over a temperature range of 1200–1450°C. A common increasing trend is displayed for all CeO_2_-Y-TZP compositions. The addition of CeO_2_ was beneficial in aiding densification as the increase in density was proportional to the increasing sintering temperature. The figure shows that samples with CeO_2_ content >0.3 wt% achieved much higher values of densities as compared to samples of lower amount and undoped samples. The highest value of density was recorded by the samples with 0.5 wt% CeO_2_, which is approximately 97% of the theoretical value (6.09 g/cm^3^) [33]. A significant reduction in density values was observed at higher temperatures, a phenomenon probably attributed to the grain growth. Besides that, the decrease could also be associated with agglomeration of the CeO_2_-Y-TZP particles during the preparation and sintering processes [34].

**Figure 2:**
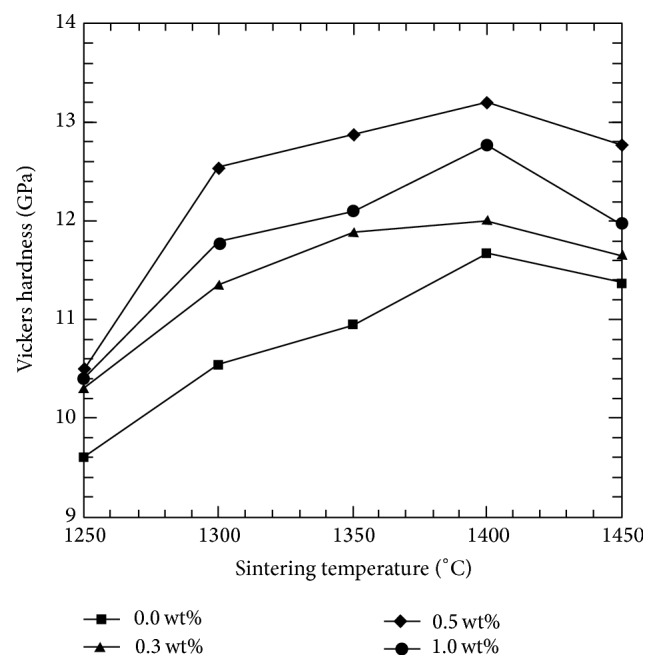
Effect of sintering temperature and CeO_2_ addition to the Vickers hardness of Y-TZP.

The effect of sintering temperatures and CeO_2_ doped samples on the Vickers hardness of Y-TZP is shown in Figure 2. The undoped sample recorded a hardness value of 9.6 GPa (978.9 HV). When sintered at 1250°C, the doped samples showed higher hardness values at 1250°C but was still below the theoretical value. Generally, all samples showed a similar trend with increasing sintering temperature profiles. The highest value recorded was 13.2 GPa (1346 HV) at a sintering temperature of 1400°C, before facing a drop in value with further sintering at 1450°C. Overall, from observation it was found that samples with 0.5wt % and 1.0 wt% dopant addition sintered at temperatures >1300°C achieved hardness values higher than the theoretical Vickers hardness of Y-TZP. The decrease in hardness at high temperatures (>1400°C) can probably be associated with the increased proportion of transformable tetragonal phase and associated pseudoplasticity [35].

**Figure 3:**
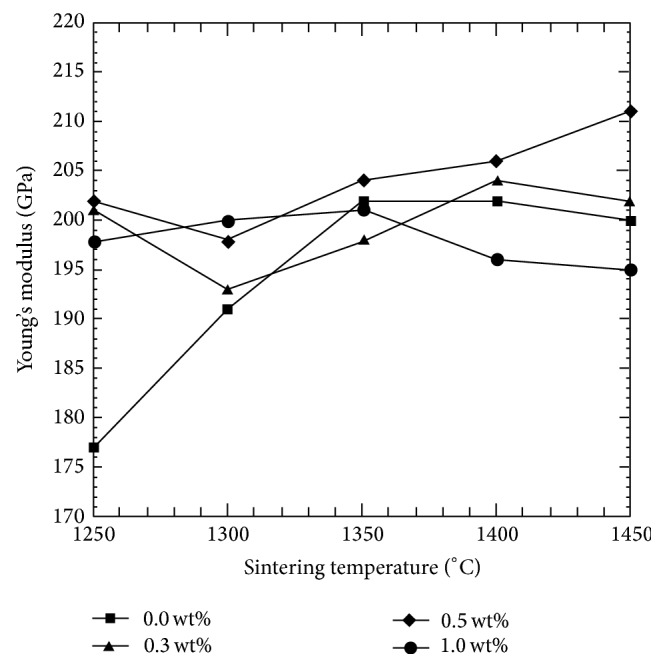
Effect of sintering temperature and CeO_2_ addition to Young's modulus of Y-TZP.

The influence of the addition of CeO_2_ on Young's modulus of the sintered sample is depicted in Figure 3. At 1250°C, all doped samples reached almost the theoretical value of Young's modulus (210 GPa) [36] whereas the undoped Y-TZP was low (~177 GPa). *E* values of ≥ 200 GPa were achieved at sintering temperatures ≥ 1300°C. The 0.3 wt% CeO_2_-Y-TZP doped sample shows a decline in *E* value from 1350°C onward, and in contrast 0.5 wt% CeO_2_-Y-TZP kept increasing up till 1450°C and attained the highest value of ~211 GPa. An inversly proportional relationship exists between the bulk density and Young's modulus as a lower density results in a higher elasticity in the material. It can be inferred that the bulk density is an important parameter governing the matrix stiffness of Y-TZP. Similar results were seen in work from other researchers [37, 38].

**Figure 4:**
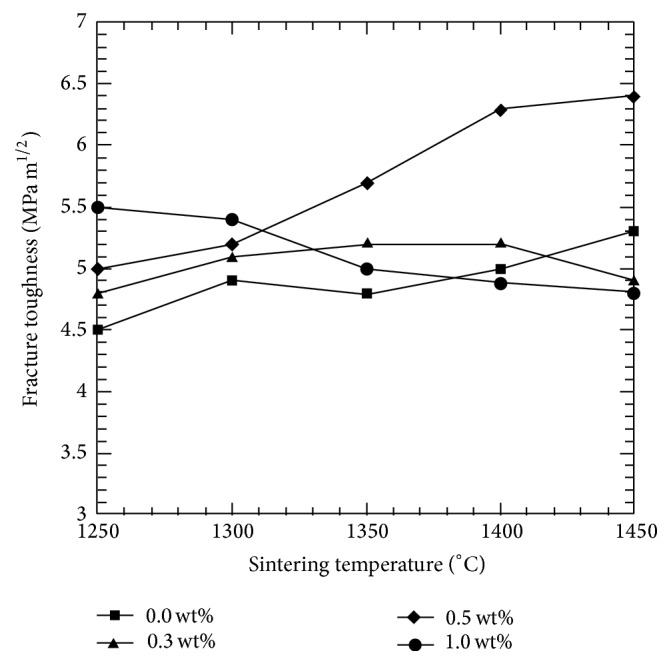
Effect of sintering temperature and CeO_2_ addition to the fracture toughness of Y-TZP.

The influence of the varying amounts of CeO_2_ and various sintering temperatures on the fracture toughness was seen in Figure 4. It was clearly seen that the addition of CeO_2_ was beneficial in enhancing the fracture toughness of Y-TZP. At 1250°C, the undoped sample only reached the fracture toughness value of 4.5 MPam^1/2^ while doped samples achieved the higher toughness ranging from 4.8 MPam^1/2^ to 5.5 MPam^1/2^. All samples showed improvement in toughness when sintered ≥1300°C, except for the 0.3 wt% doped samples, which in contrast displayed a decreasing trend up till 1450°C. Sintering at higher temperature (≥1300°C) seems to have an outstanding effect on the fracture toughness of Y-TZP, especially with the addition of 0.5 wt% CeO_2_. The highest value of fracture toughness recorded was 6.4 MPam^1/2^ for 0.5 wt % CeO_2_ addiition at 1400°C. The high fracture toughness of CeO_2_-Y-TZP sample could be attributed to the resistance of the material to crack propagation and also the grain growth [27].

**Figure 5:**
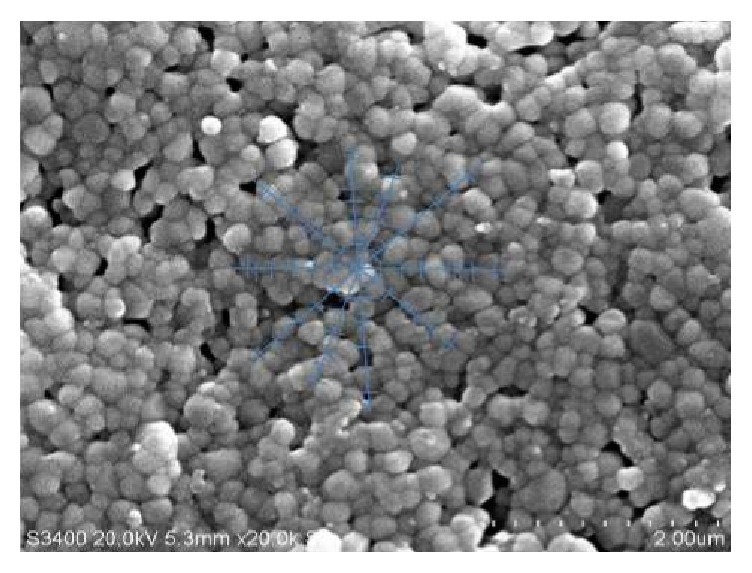
SEM imaging of the average grain intercept method for 0.3 wt% CeO_2_-Y-TZP at 1400°C.

**Figure 6:**
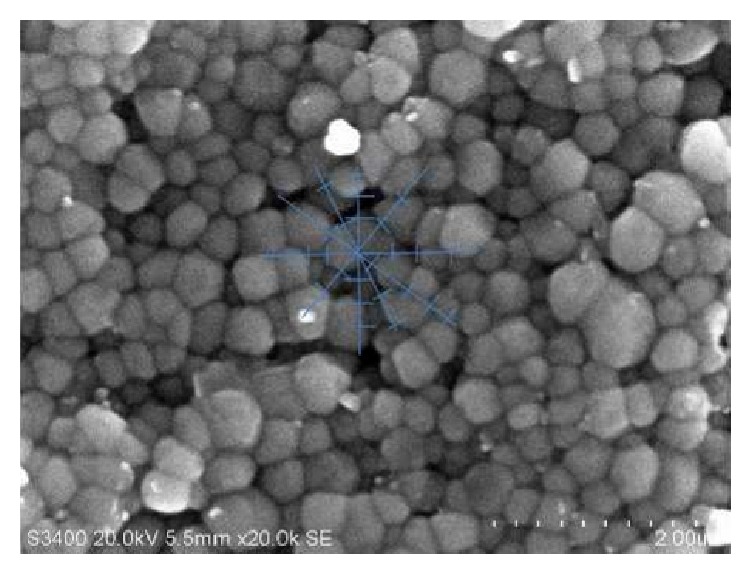
SEM imaging of the average grain intercept method for 0.5 wt% CeO_2_-Y-TZP at 1400°C.

Figures 5 and 6 illustrate the effect of the addition of 0.3 wt% and 0.5 wt% CeO_2_ on the microstructure of the ceramic. The 0.5 wt% doped sample revealed a microstructure that is more homogenous than the microstructure of the 0.3 wt% CeO_2_ doped specimens. The average grain intercept is determined from two of the best samples (polished) with different dopant content sintered at 1400°C. It is seen that the samples containing 0.3 wt% CeO_2_ have a bigger grain size with an average value of 2.13 *μ*m whereas the samples with 0.5 wt% CeO_2_ have a smaller grain size value of 1.586 *μ*m. Grain growth was proportional to the amount of dopant added.

## 5. Conclusion

The current work shows that adding CeO_2_ to Y-TZP has proved to be beneficial. The bulk density was found to be higher with a value of ~5.9 g/cm^3^ (~97% of the theoretical density), with the addition of up to 0.5 wt% CeO_2_ when sintered at 1400°C. Doping CeO_2_ into Y-TZP also enhanced other mechanical properties. All samples with 0.5 wt% CeO_2_ produced the highest values for mechanical properties; the Vickers hardness was increased to ~13.2 GPa; Young's modulus was enhanced to ~211 GPa; fracture toughness was increased to ~6.4 MPam^1/2^. Also, sintering temperature had an important role in determining the mechanical properties and microstructure of the material as most increment in values occurred at temperatures ≥ 1300°C.
